# Supplementary figures and images for: Hrq1/RECQL4 regulation is critical for preventing aberrant recombination during DNA intrastrand crosslink repair and is upregulated in breast cancer
Source: PLoS Genet. 2022 Sep 20;18(9):e1010122. doi: 10.1371/journal.pgen.1010122 (PMC9488787; doi:10.1371/journal.pgen.1010122)

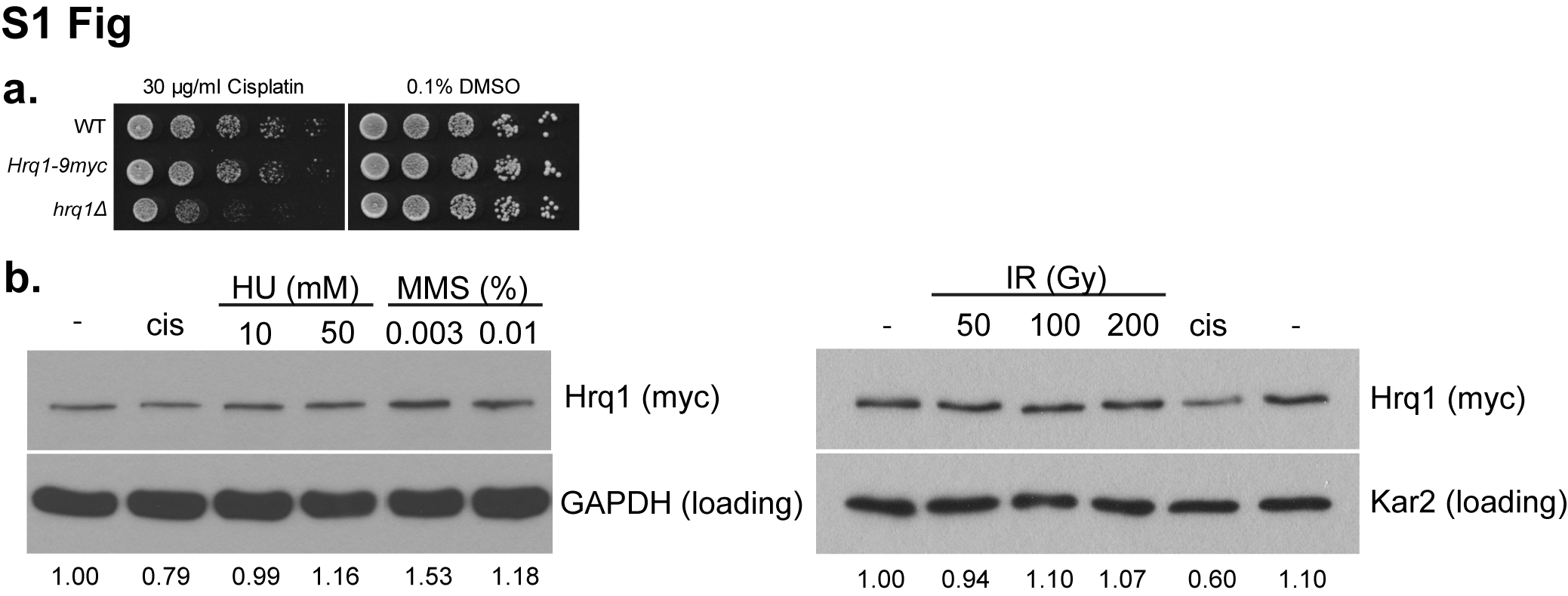

Supplement: S1 Fig — (A) Hrq1-9myc cells displayed similar cisplatin sensitivity compared to WT cells. The indicated yeast strains were five-fold serial diluted onto SC medium containing DMSO and/or SC medium containing the indicated amount of cisplatin. The plates were photographed after 2 days of incubation at 30°C in the dark. (B) Hrq1 protein level is unchanged with HU and MMS treatment. Hrq1-9myc cells were grown to log phase and then treated with indicated agents for two hours before being collected for TCA prep. Cisplatin was used at 100 μg/ml (Raw densitometry data in S11 Data). (TIF) [file pgen.1010122.s001.tif]

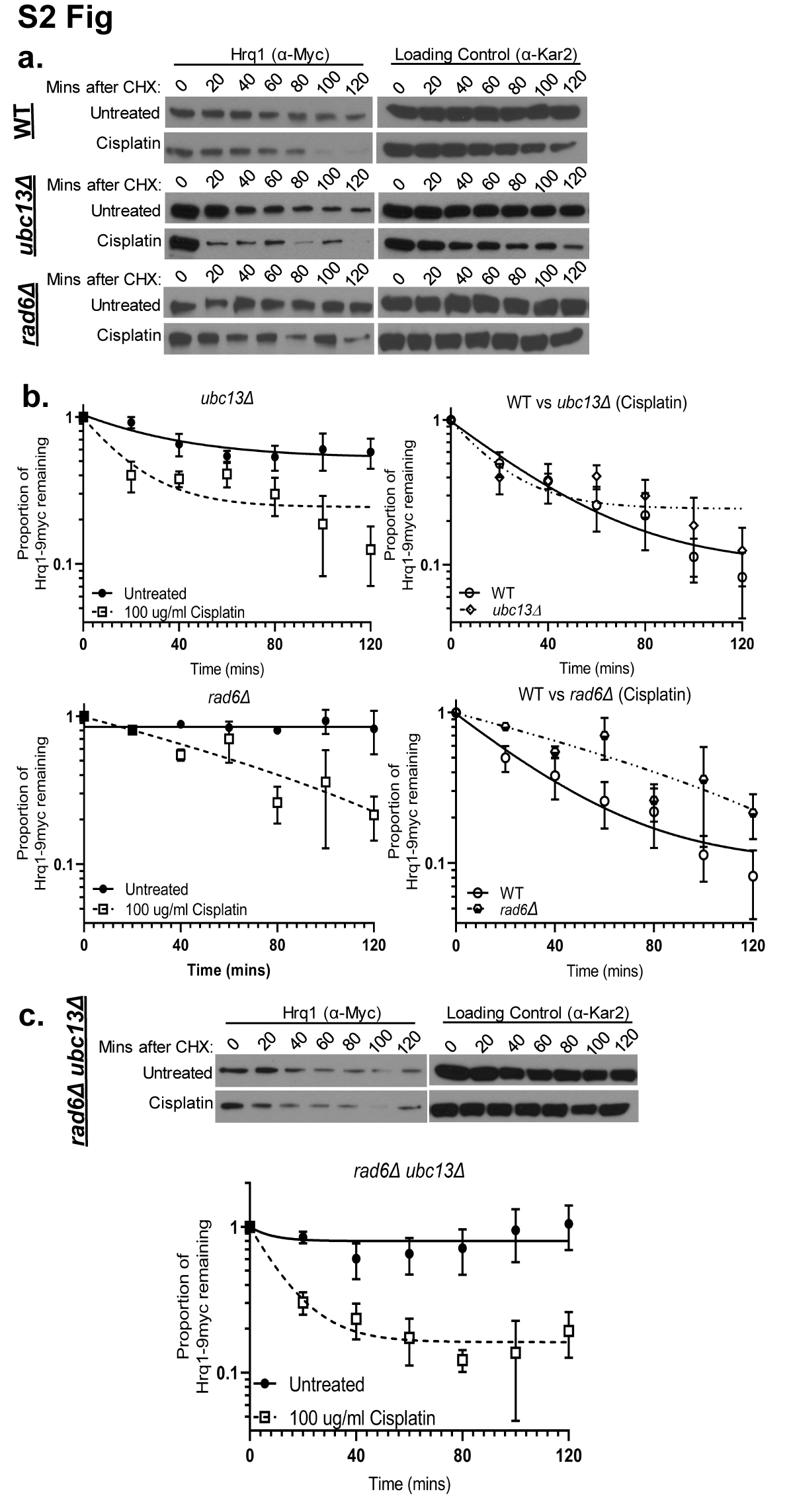

Supplement: S2 Fig — (A) Hrq1-9xMYC expressing ubc13Δ or rad6Δ cells were incubated with cycloheximide in the presence or absence of 100 μg/ml cisplatin and/or 0.1% DMSO. Protein extracts were analyzed by western blot for Hrq1 protein levels (α-MYC) or a loading control, Kar2 (α-Kar2), at the indicated time points. (B) Quantification of the proportion of Hrq1 remaining relative to time 0 (before CHX addition) and the loading control, Kar2, are plotted on the graph in log scale from ubc13Δ or rad6Δ cells. Each experiment was performed in triplicate with standard error plotted (Raw densitometry data in Sheets I-N in S1 Data). WT cisplatin treated time course is replotted from Fig 1C, for direct comparison to ubc13Δ or rad6Δ cisplatin treated cells. It is important to note that Hrq1 and the loading control, Kar2, were analyzed from the same gels to account for pipetting errors. Since Hrq1 is not as abundant as the loading control, there is a limitation for the densitometry analysis. (C) Cycloheximide chase was performed as indicated in (A) however in a rad6Δ ubc13Δ background (Raw densitometry data in Sheets O-Q in S1 Data). (TIF) [file pgen.1010122.s002.tif]

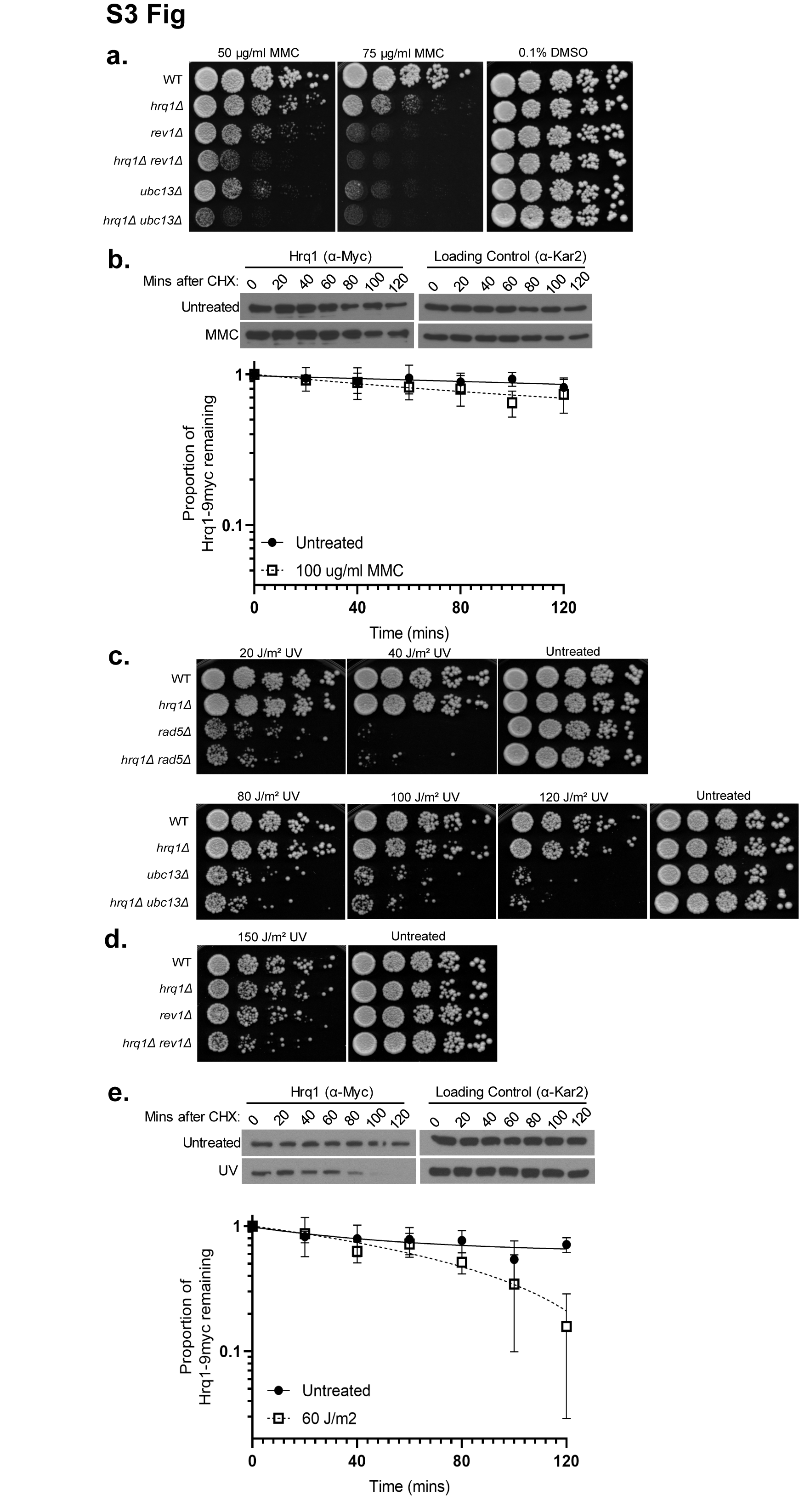

Supplement: S3 Fig — (A) Hrq1 functions in a different pathway as Rev1 and Ubc13 to repair ICL. The indicated yeast strains were five-fold serial diluted onto YPD medium containing DMSO and/or YPD medium containing the indicated amount of MMC. The plates were photographed after 2 days of incubation at 30°C in the dark. (B) Hrq1 protein levels does not decreased upon 100 μg/ml MMC treatment. Exponentially growing cells with Hrq1-9xMYC were incubated with cycloheximide in the presence or absence of 100 μg/ml MMC and/or 0.1% DMSO. Protein extracts were analyzed by western blot for Hrq1 protein levels (α-MYC) or a loading control, Kar2 (α-Kar2) at the indicated time points. Quantification from three separate experiment is shown, with mean and SEM graphed (Raw densitometry data in Sheets S-U in S1 Data). (C) Hrq1 functions in the same pathway as Rad5 and Ubc13 to repair intrastrand adducts. The indicated yeast strains were five-fold serial diluted onto YPD medium before being treated with the indicated dosages of UV-C. The plates were photographed after 2 days of incubation at 30°C in the dark. (D) Hrq1 functions in different pathway as Rev1 to repair intrastrand adducts. The indicated yeast strains were five-fold serial diluted onto YPD medium before being treated with the indicated dosage of UV-C. The plates were photographed after 2 days of incubation at 30°C in the dark. (E) Hrq1 protein levels decreased following UV-C treatment. Exponentially growing Hrq1-9xMYC cells were incubated with cycloheximide then treated with indicated dosage of UV-C. Protein was extracted similarly to (B). Quantification is from three separate experiments with mean and SEM graphed (Raw densitometry data in Sheets V-X in S1 Data). (TIF) [file pgen.1010122.s003.tif]

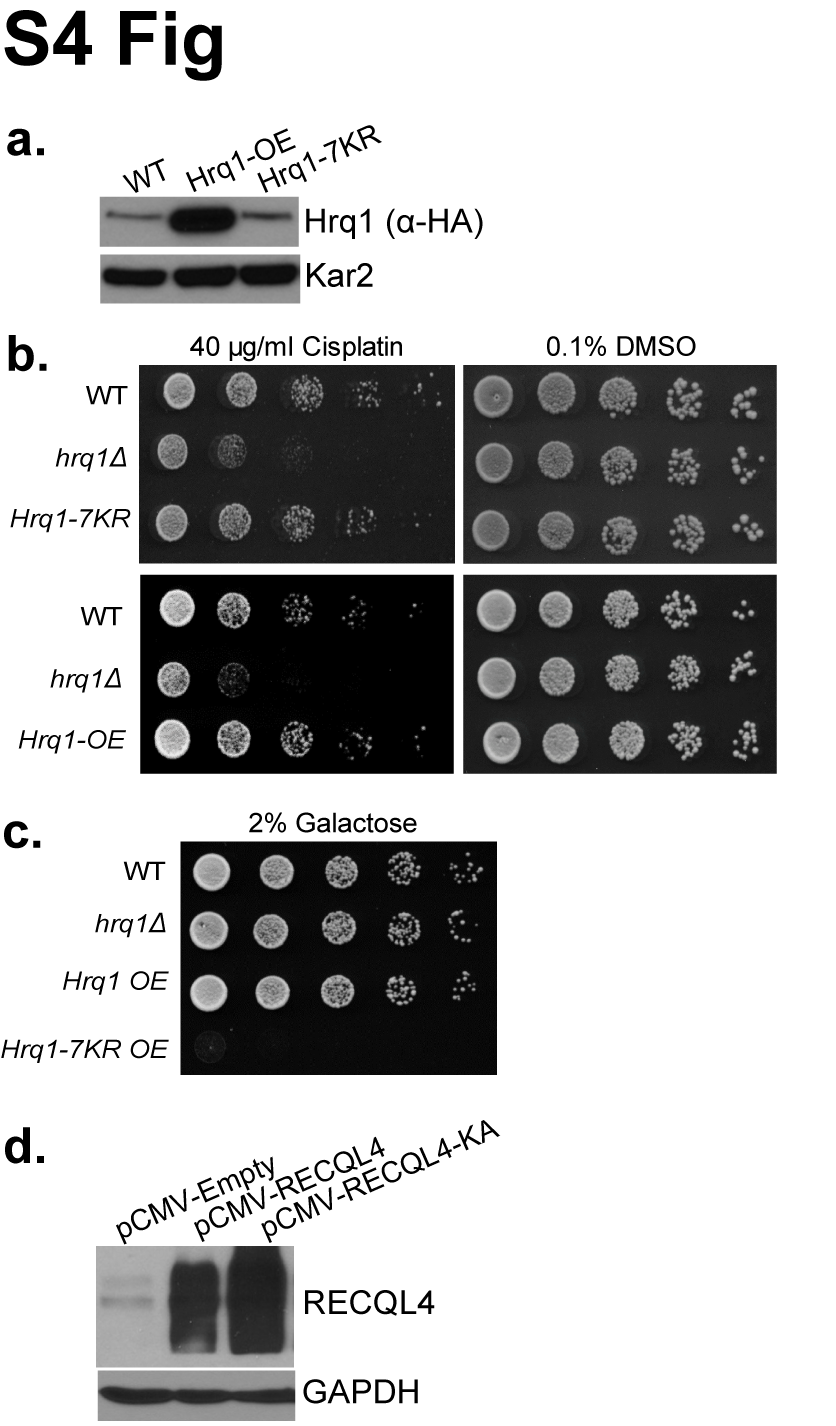

Supplement: S4 Fig — (A) Hrq1-7KR protein levels are similar to WT in basal conditions. The indicative strains were grown overnight in 2% galactose, subsequently TCA was performed and western was run to determined protein level. (B) Stabilization or overexpression of Hrq1 does not lead to increased cisplatin sensitivity. The indicated yeast strains were five-fold serial diluted onto SC medium containing DMSO and/or SC medium containing the indicated amount of cisplatin. The plates were photographed after 2 days of incubation at 30°C in the dark. Plates that were used with Hrq1-OE (GAL-HRQ1, galactose inducible/dextrose repressible promoter) strains were made with galactose instead of glucose medium. (C) Overexpression of the Hrq1-7KR mutant results in cell lethality. The indicated yeast strains were grown overnight in SC medium containing raffinose. Subsequently they were five-fold serial diluted onto galactose SC plates. The plates were photographed after 2 days of incubation at 30°C in the dark. (D) pCMV-RECQL4-KA promotes similar overexpression compared to pCMV-RECQL4. Protein extracts from cells 3 days post-transfection. Endogenous RECQL4 was detected using α-RECQL4, GAPDH served as a control. (TIF) [file pgen.1010122.s004.tif]
