# Supplementary material for: Hrq1/RECQL4 regulation is critical for preventing aberrant recombination during DNA intrastrand crosslink repair and is upregulated in breast cancer
Source: PLoS Genet. 2022 Sep 20;18(9):e1010122. doi: 10.1371/journal.pgen.1010122 (PMC9488787; doi:10.1371/journal.pgen.1010122)
Supplement: Attachment — Submitted filename: Revision2 Reviews PLoS Genetics.pdf [file pgen.1010122.s019.pdf]

Reviewer #1: Despite the revision, the results regarding ubiquitination of Hrq1 still appear to be too preliminary, and many of the points I have raised have not been addressed.

Reviewer 1:
1. In Fig. 1 c, d; Fig.2 a, b; Sup. Fig. 2
I assumed that authors include representative images in figures. However, many images in figures are overexposed and did not match well with graphs. Based on description in Materials and Methods, authors seemed to use densitometry analysis of scanned Western blots images for quantitation. Especially, in this case, images should not be overexposed.
As the reviewer pointed out, the images in these figures are from three or more experiments and are representative images. Unfortunately, since Hrq1 is not well expressed relative to the loading control, the loading control can appear over-exposed since the images are quantitated from the same blots. Hrq1 is expressed from its endogenous locus and promoter. We have now included less exposed images where possible to alleviate this technical issue.

• Densitometry analysis of overexposed images is not acceptable for quantitation. Overexposed images of the Western blotting cannot represent the actual difference in protein levels. As quantitation has not been done properly, these experiments should be repeated. It is not necessary to use the same exposure time in the control blot (GAPDH or Kar2). You have to use short exposed images to avoid saturated bands. Alternatively, you can use the ChemiDoc imager detecting chemiluminescence for quantitation. Again, saturated bands should be avoided.

We respectfully disagree with the reviewer on this important point. Because Hrq1 is not abundant, we had to load a sufficient amount of lysate to visualize Hrq1 and this results in more Kar2 or GAPDH being loaded than ideal. Otherwise, we would have to run two separate gels but then we wouldn't be able to account for technical errors in loading. We have now noted this limitation in the manuscript in the figure legend.

Lines 991-993 "It is important to note that Hrq1 and the loading control, Kar2, were analyzed from the same gels to account for pipetting errors. Since Hrq1 is not as abundant as the loading control, there is a limitation for the densitometry analysis.".

2. In Fig. 1b.
You used only single dose of chemicals in this experiment. Did you use enough and comparable dose of other chemicals in this experiment? I even cannot find what dose of chemicals and irradiation you have used.
We agree with the reviewer, and we inadvertently left off the doses analyzed, which are now included. In addition, we have now performed additional experiments analyzing multiple doses (New Supplemental Figure 1b).
New Supplemental Figure 1b Legend
Steady state levels of Hrq1 upon HU and MMS treatment. Hrq1-9-Myc expressing cells were grown to early log phase and then treated with indicated DNA damaging agent for two hours

(10 and 50 mM HU, 0.003% and 0.01% MMS, or 100 µg/ml cisplatin) or IR exposed (50, 100, 200 Gy) before protein analysis of whole cell extracts by western blot. GAPDH or Kar2 was used as a loading control. Quantification of Hrq1 (anti-Myc antibody) relative to GAPDH or Kar2 is shown below and normalized to the untreated Hrq1-9-Myc control.

• Question is whether you used enough concentration of other chemicals or not. The new supplemental figure 1b is not helpful because it shows the results using lower concentration of HU and MMS than the concentration used in Fig 1b.

We apologize for not fully understanding your critique. The original doses of HU (100 mM), MMS (0.03%), and IR (100 Gy) used in the first submission are already quite high and so increasing the doses further will likely result in cellular death and other indirect effects. In that scenario, it is difficult to decipher if the effects we observe are pleiotropic and therefore we used a lower range to access the effects of different DNA damaging agents on Hrq1 protein levels.

3. In Fig. 1e and Sup Fig. 1b
Patterns of Hrq1 ubiquitination are different in these reverse IP experiments. Smeared pattern of Hrq1 was found in Ub pulldown, but Hrq1 pull down made discrete single band. Why?

We agree with the reviewer that there were differences in the ubiquitin partner depending upon whether ubiquitin itself or Hrq1 was immunoprecipitated. An important difference between these experiments is that the Hrq1 pull down used 25% of the yeast culture compared to the ubiquitin pull down. Therefore, one possibility is that more Hrq1 species can be visualized. Furthermore, the ubiquitin pulldown relies on massive over-expression of ubiquitin and perhaps that creates differences compared to analyzing the endogenous Hrq1 protein that is not over-expressed. We have made a note of this in the Results.

• I hardly believe that the amount of extracts makes different pattern of ubiquitinated protein precipitates. If you think different amount of extracts make a difference, this experiment should be repeated using the same amount of extracts.

As reviewer 3 suggested, we have now removed this data from the manuscript.

5. In Fig. 3a
Alpha factor arrested cells are not real G1 cells. Alpha factor arrests cell cycle before start of cell cycle at G1, and expression of many genes involved in cell proliferation can be reduced in alpha factor arrested cells. You can analyze the G1 cells in the next cell cycle or you can use other methods for cell synchronization such as nocodazole arrest and release to make sure that changes in the level of proteins indeed occur in G1.

We respectfully disagree with the reviewer as alpha factor is extensively used to arrest cells in G1. Regardless, our main conclusion is to demonstrate that Hrq1 expression peaks before Clb2 expression.

• You described in the manuscript that you did this experiment to see whether its protein levels are cell cycle regulated, and Hrq1 protein levels peak in S/G2. While Clb2 clearly shows a peak, Hrq1 protein level begins to increase and then reaches the plateau. Unless you show decrease in Hrq1 levels during the cell cycle, you cannot say its level peaks in S/G2.

As the reviewer suggests, we have now changed the text to indicate that Hrq1 protein levels begin to increase in S/G2 and then reach a plateau.

Lines 194-196 *"Consistent with a role during replication, Hrq1 protein levels begin to increase in S/G2 at 40 min after alpha factor release and then plateaus whereas Clb2 protein peaks starting at 60 min (**Figure 3a**)."*.

7. In Sup Fig. 3b
Again, you have to use comparable dose of MMC in this experiment. Based on sensitivities of yeast cells to cisplatin and MMC in Fig. 3 and Sup. Fig. 3, yeast cells are much more sensitive to cisplatin than MMC at the same concentration. Therefore, the result obtained by using the same concentration of MMC is not conclusive.
Unfortunately, we cannot directly compare doses of different damaging agents as they cause different types of DNA damage and their effects not necessarily the same on hrq1Δ cells as observed by the different sensitivity. However, we have provided multiple cisplatin and MMC doses for analysis here (Supplemental Figure 3a).

• When you analyzed the stability of Hrq1, you used 100 ug/ml of cisplatin which is more than 3 times higher than the highest concentration used in the repair assay (30 ug/ml). As you used very high concentration of cisplatin, the effect shown here could be indirect. The concentration of MMC used in stability test (100 ug/ml) is only 1.3 times higher than the concentration used in repair assay (75 ug/ml). What would be the stability of Hrq1 if you use a higher concentration of MMC?

It is possible that we would observe a reduction in Hrq1 protein levels upon a high MMC dose. We have incorporated this caveat into the text.

Lines 259-262 *"While it is possible that higher doses of MMC may result in Hrq1 degradation, together our genetic and molecular results suggest that Hrq1 function and regulation in response to ICLs is indeed distinct from intrastrand crosslinks."*.

8. In Figure 4c
The levels of Hrq1 in WCE are different. If you want to directly compare the intensities of

ubiquitinated proteins in IP materials, you have to use the same amount of extracts containing the same amount of target proteins.
We agree with the reviewer that the levels of Hrq1 in WCE are different either due to pipetting error or to degradation from cisplatin exposure. To account for the difference in the WCE, we normalized the IP to the WCE, which mitigates any of these technical differences.

• The difference between wild type and mutant pull-down is too small. Considering the many steps required for ubiquitination assay, this small difference could be simple variation between experiments. It is difficult to reach any conclusion unless a clear and significant difference is observed. In addition, you have to show the Western blot of Ub to show that a similar amount of ubiquitins were pulled-down in this experiment.

We have now removed this data from the manuscript.

Reviewer #2: I appreciate the care with which the authors attempted to address all the reviewers' comments in their revisions. Many of the key issues raised by the reviewers have been addressed in a satisfactory manner, improving the quality of the manuscript, and strengthening its conclusions. Several key issues remain.

Major Comments:

Lines 205-208, Figure 3A: I appreciate the inclusion of the quantitation of the Western blots for this figure. As observed from the blots and quantitation, Clb2 clearly is most strongly expressed at 60 minutes, after which its expression decreases. The same is not true for Hrq1. According to the quantitation, it appears that Hrq1 expression peaks at 80 or 100 minutes, with very little change in expression from 40 to 120 minutes. It is not clear from this analysis when Hrq1 expression will again return to the low levels observed at the beginning of the time course. The claims about this data should be softened, as they do not appear to support the conclusion that Hrq1 protein levels peak at 40 minutes.

We agree with the reviewer and as suggested by Reviewer 1 we have changed the text to indicate that Hrq1 protein levels begin to increase in S/G2 and then reach a plateau.

Lines 194-196 *"Consistent with a role during replication, Hrq1 protein levels begin to increase in S/G2 at 40 min after alpha factor release and then plateaus whereas Clb2 protein peaks starting at 60 min (**Figure 3a**)."*.

Figures 5C and 5D: The response that Lu et al. 2017 Nat Comm has the correct siRNA control to show that expression of siRNA-resistant RECQL4 restores the wild-type phenotype is well-taken. An siRNA-resistant REV1 plasmid could still be created so that the authors can perform this important control. Several companies (e.g. Twist Biosciences) now will clone up to 5 kbp into a plasmid of your choice at a reasonable cost. However, I appreciate that the authors summed up

their results in a careful way (lines 397-400).

We agree with the reviewer that it is an important control and we worked hard to both create and obtain the plasmid for the revision, which took 4 months and were sent truncated reagents and were independently unsuccessful with cloning. In the interest of time and because of the other compelling data that is consistent with these findings from multiple angles and model systems, we had to forgo this experiment.

Minor Comments:

Lines 115-117: As stated previously, analysis of RAD51 foci counts alone is not sufficient evidence to conclude that there is increased recombination. Increased RAD51 foci could also indicate failure to resolve recombination intermediates. I appreciate the authors mentioning these possibilities in lines 413-415.

Corrected.

Lines 115-117 *"Moreover, overexpression of RECQL4 results in increased RAD51 foci, which is dependent on its helicase activity."*

Lines 345-346: To incorporate the result that both HR and SSA are increased, these lines might be rephrased to say, "These results suggest high levels of Hrq1 following cisplatin exposure results in increased homology-directed repair…" or something to this effect.

Corrected.

Lines 315-316 *"These results suggest high levels of Hrq1 following cisplatin exposure results in increased homology-directed repair."*

Reviewer #3: In this revised manuscript the authors have provided additional data and clarifications to support their assertion that yHrq1 and hRECQL4 promote error-free, HR-dependent bypass of lesions induced by genotoxins that general induce DNA intrastrand cross-links. They also extend their analysis of a conserved mechanism for regulating yHrq1 and hRECQL4 through proteasome-dependent degradation to show that yHrq1 is also degraded upon UVC treatment. The conclusions are generally well founded, and the authors have allayed several of my concerns. However, I still have concerns as to quality/utility of some of the data.

1) I am still unconvinced that Hrq1 undergoes ubiquitylation in response to cisplatin-induced DNA damage. While this model is certainly plausible, the His-Ub pulldown experiments (despite the authors' efforts) sill lack appropriate specificity controls and no evidence is provided to

support the conclusion that slower migrating species observed in His-Ub pulldown experiments correspond to ubiquitylated species. Additionally, the reported enhanced recovery of the ubiquitylated species is not internally controlled (instead relying on comparison with the WCE lane) and could therefore arise as a loading artifact. This is especially problematic since both the putative ubiquitylated forms and unmodified Hrq1 are recovered in these experiments and the recovery of both forms appears to be increased in response to cisplatin. Results indicating a failure to recover ubiquitylated species in the presence of the Hrq1-7KR mutated protein when pulling down Hrq1-7KR OR His-Ub (4c and S1d) are also uninformative since these experiments do not appear to have been performed in parallel with experiments using wild-type Hrq1 for comparison. Overall, I strongly recommend omitting data relating to the ubiquitylation of Hrq1 since the reported experiments are unconvincing and detract from the rest of the manuscript (which is compelling).

As the reviewer suggested, we have now removed this data from the manuscript.

2) Data demonstrating that the K164R mutant stabilizes Hrq1 upon cisplatin treatment would enhance that paper. As presented in S2d (without a wild-type PCNA condition for reference), however, the effect of the K164R mutation cannot be assessed.

We have now removed this data from the paper.
