# Supplementary material for: Hrq1/RECQL4 regulation is critical for preventing aberrant recombination during DNA intrastrand crosslink repair and is upregulated in breast cancer
Source: PLoS Genet. 2022 Sep 20;18(9):e1010122. doi: 10.1371/journal.pgen.1010122 (PMC9488787; doi:10.1371/journal.pgen.1010122)
Supplement: Attachment — Submitted filename: Response to Reviewer Comments.pdf [file pgen.1010122.s018.pdf]

Response to Reviewer Comments

Reviewer 1:

1. In Fig. 1 c, d; Fig.2 a, b; Sup. Fig. 2
I assumed that authors include representative images in figures. However, many images in figures are overexposed and did not match well with graphs. Based on description in Materials and Methods, authors seemed to use densitometry analysis of scanned Western blots images for quantitation. Especially, in this case, images should not be overexposed.

As the reviewer pointed out, the images in these figures are from three or more experiments and are representative images. Unfortunately, since Hrq1 is not well expressed relative to the loading control, the loading control can appear over-exposed since the images are quantitated from the same blots. Hrq1 is expressed from its endogenous locus and promoter. We have now included less exposed images where possible to alleviate this technical issue.

2. In Fig. 1b.
You used only single dose of chemicals in this experiment. Did you use enough and comparable dose of other chemicals in this experiment? I even cannot find what dose of chemicals and irradiation you have used.

We agree with the reviewer, and we inadvertently left off the doses analyzed, which are now included. In addition, we have now performed additional experiments analyzing multiple doses (**New Supplemental Figure 1b**).

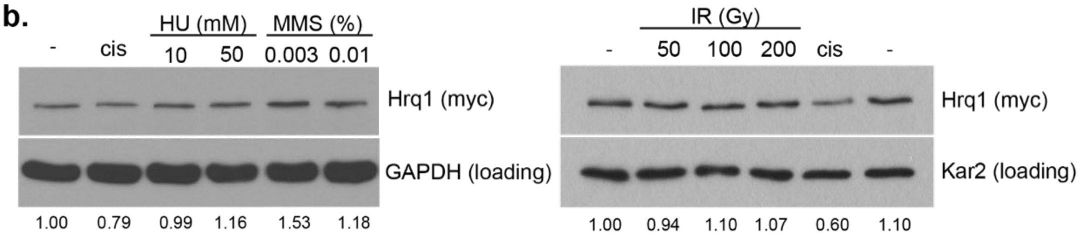

New Supplemental Figure 1b Legend
Steady state levels of Hrq1 upon HU and MMS treatment. Hrq1-9-Myc expressing cells were grown to early log phase and then treated with indicated DNA damaging agent for two hours (10 and 50 mM HU, 0.003% and 0.01% MMS, or 100 µg/ml cisplatin) or IR exposed (50, 100, 200 Gy) before protein analysis of whole cell extracts by western blot. GAPDH or Kar2 was used as a loading control. Quantification of Hrq1 (anti-Myc antibody) relative to GAPDH or Kar2 is shown below and normalized to the untreated Hrq1-9-Myc control.

3. In Fig. 1e and Sup Fig. 1b
Patterns of Hrq1 ubiquitination are different in these reverse IP experiments. Smeared pattern of Hrq1 was found in Ub pulldown, but Hrq1 pull down made discrete single band. Why?

We agree with the reviewer that there were differences in the ubiquitin partner depending upon whether ubiquitin itself or Hrq1 was immunoprecipitated. An important difference between these experiments is that the Hrq1 pull down used 25% of the yeast culture compared to the ubiquitin pull down. Therefore, one possibility is that more Hrq1 species can be visualized. Furthermore, the ubiquitin pulldown relies on massive over-expression of ubiquitin and perhaps that creates differences compared to analyzing the endogenous Hrq1 protein that is not over-expressed. We have made a note of this in the Results.

4. In Fig. 2a
Hrq1 appeared to be already unstable in Rad16 deleted cells even in the absence of cisplatin. Why? Again, I do not see much difference in Hrq1 stability of wt and rad16 deleted cells in these images, while graphs shows differently.

We agree with the reviewer that we should explain these results more clearly. Hrq1 is both cell cycle regulated and regulated upon exposure to intrastrand adducts. Therefore, there may be multiple independent modes of regulation. We find that deleting *RAD16* stabilized Hrq1 protein levels upon cisplatin exposure specifically (**Figure 2**). There could be other ubiquitin ligases that regulate Hrq1 protein levels that are DNA damage independent. We have clarified this point in the Results.

5. In Fig. 3a
Alpha factor arrested cells are not real G1 cells. Alpha factor arrests cell cycle before start of cell cycle at G1, and expression of many genes involved in cell proliferation can be reduced in alpha factor arrested cells. You can analyze the G1 cells in the next cell cycle or you can use other methods for cell synchronization such as nocodazole arrest and release to make sure that changes in the level of proteins indeed occur in G1.

We respectfully disagree with the reviewer as alpha factor is extensively used to arrest cells in G1. Regardless, our main conclusion is to demonstrate that Hrq1 expression peaks before Clb2 expression.

6. In Fig. 3f
Differences in cisplatin sensitivity between rev1 deletion and hrq1 and rev1 deletion are so marginal, and only found in a single plate (20 ug/ml cisplatin). I am very skeptical to make any conclusion based on these observations.

We agree with the reviewer that the synthetic sickness observed between *hrq1*Δ *rev1*Δ double mutant is subtle but highly reproducible. We provide additional evidence that siRNA knockdown of human RECQL4 also exhibits a subtle, but highly reproducible, growth defect upon siRNA knockdown of REV1. These results are consistent with Groocock et al 2012, who showed that fission yeast *hrq1*Δ cells exhibit more spontaneous mutations. Together, these data support our observations here (**Figure 5c, 5d**) and we now include these findings from Groocock in the paper.

7. In Sup Fig. 3b
Again, you have to use comparable dose of MMC in this experiment. Based on sensitivities

of yeast cells to cisplatin and MMC in Fig. 3 and Sup. Fig. 3, yeast cells are much more sensitive to cisplatin than MMC at the same concentration. Therefore, the result obtained by using the same concentration of MMC is not conclusive.

Unfortunately, we cannot directly compare doses of different damaging agents as they cause different types of DNA damage and their effects not necessarily the same on *hrq1*Δ cells as observed by the different sensitivity. However, we have provided multiple cisplatin and MMC doses for analysis here (**Supplemental Figure 3a**).

8. In Figure 4c
The levels of Hrq1 in WCE are different. If you want to directly compare the intensities of ubiquitinated proteins in IP materials, you have to use the same amount of extracts containing the same amount of target proteins.

We agree with the reviewer that the levels of Hrq1 in WCE are different either due to pipetting error or to degradation from cisplatin exposure. To account for the difference in the WCE, we normalized the IP to the WCE, which mitigates any of these technical differences.

9. In Figure 5c
You need to show the cisplatin sensitivity of double knock-down of RecQL4 and any one functioning in error-free PRR pathway to suggest the role of RecQL4 in the error-free PRR.

We agree with the reviewer that double knock-down of RecQL4 and a gene functioning in the error-free PRR pathway would be very informative. We attempted to knockdown both HLTF and SHPRH, the homologs of Rad5. However, the results were inconclusive. Knockdown of *HLTF* alone leads to decreased cellular proliferation even in the absence of damage, which confounded our results when treated with cisplatin. On the other hand, knockdown of *SHPRH* seems to result in cisplatin resistance (**Reviewer Figure 1**). More extensive analysis is needed to elucidate this complex pathway and is beyond the scope of the current paper. Therefore, we have softened the language discussing RecQL4 function with error-free PRR in the Results.

Reviewer Figure 1

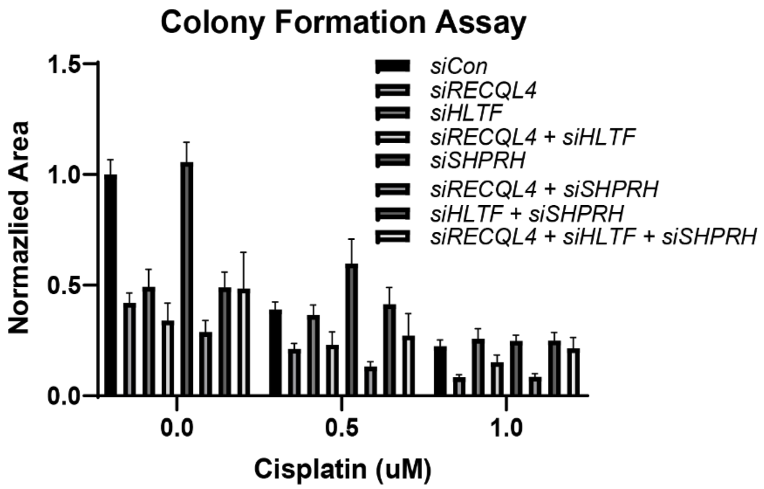

Reviewer Figure 1 Legend:
U2OS cells were transfected with either siCon, siRECQL4, siHLTF, and/or siSHPRH for 48 hours and 300 cells per well were plated in triplicate. The following day the medium was replaced with fresh medium with or without the indicated dose of cisplatin. After 24 hours, the medium was removed, the cells were washed, and fresh medium was added. The cells grew for 17 days before crystal violet staining. The experiment was repeated in triplicate with standard error plotted and the colonies were normalized to the untreated siCon.

10. In Fig. 6.
Rad51 staining is poor, and cisplatin treatment did not increase Rad51 foci in immunostaining and tail moments in comet assay even in U2OS cells. Therefore, I do not think that these assays are reliable in this condition.

We agree with the reviewer that tail moments in the comet assay did not exhibit more DSBs. Since we were focusing on bypass of replicative damage and not direct DSB repair, we did not analyze the cells at a time point where DSBs would form. Also, treatment with S1, which should result in increased DSBs, lead to increased tail moments, which indicates the assay worked as expected. We have clarified this point in the Results.

With regards to the RAD51 staining, we have now included improved image quality so that the foci are more easily visualized (**New Figure 6a**). The RAD51 foci are analyzed automatically in a blinded fashion, and this enables appropriate thresholding and background to be uniformly assessed. We have expanded upon this analysis in the Materials & Methods.

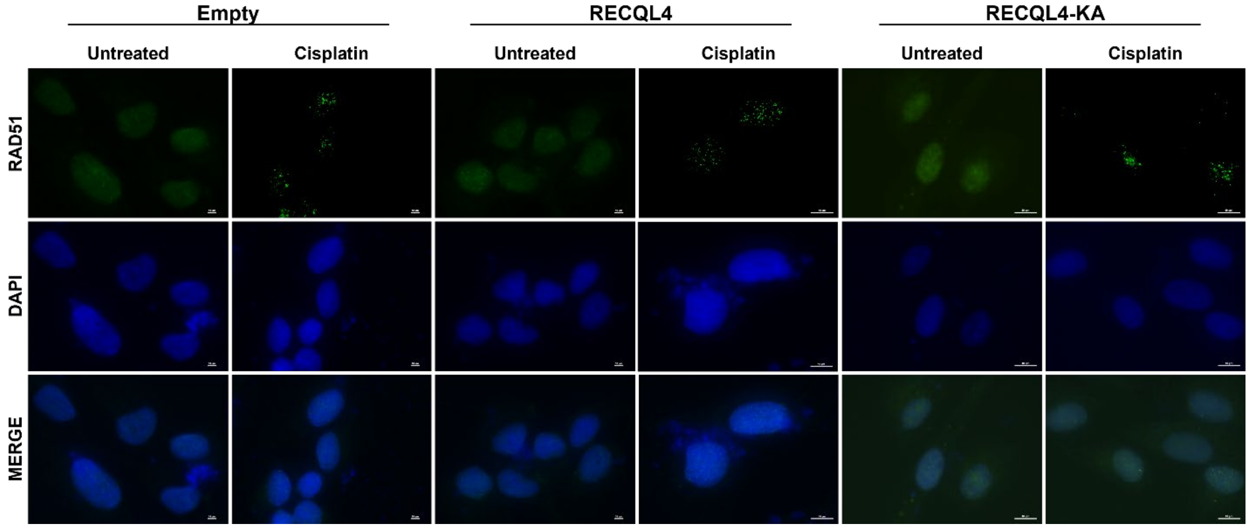

New Figure 6a Legend:
Overexpression of *RECQL4* results in increased RAD51 foci, which is dependent on its helicase activity. U2OS cells were transfected with an empty plasmid or a plasmid expressing RECQL4 or RECQL4-K508A under a CMV promoter. The cells were either mock or cisplatin treated for one hour and after a two-hour recovery, imaged for RAD51 foci or DAPI by immunofluorescence. RAD51 foci was quantified from 200 cells per condition for each experiment. The experiment was performed three to five times and the median was graphed. Representative images are shown.

Reviewer 2

Major Comments:

1) Figure 3A: Whereas it is obvious from the Western blot images provided that Clb2 peaks at 60 minutes, the same is not true for the protein of interest, Hrq1-9-Myc. Instead, Hrq1-9-Myc appears to plateau at 40 minutes. To strengthen the conclusion that Hrq1 protein levels peak at 40 minutes, I recommend quantification of the results in these blots as in Figures 1C and 2A.

We have now quantitated the western blots from **Figure 3a** and indeed Hrq1 begins to peak prior to Clb2 (**New Figure 3a**).

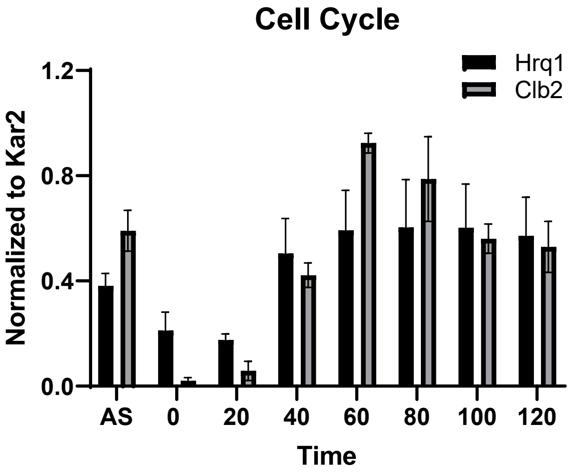

New Figure 3a Legend: Hrq1 expression peaks during S/G2. Hrq1-9xMYC expressing cells were either untreated (asynchronous, AS) or cell cycle arrested in G1 with α-factor. The α-factor arrested cells were subsequently released into fresh YPD medium (0 min) and grown for 120 min. Protein samples from the indicated time points were analyzed by western blot for Hrq1 (anti-MYC), the G2/M cyclin, Clb2, (anti-Clb2), or a loading control, Kar2 (anti-Kar2). Quantification from three separate experiment, the mean with SEM is shown. The cell cycle stage was analyzed FACS.

Between 0 and 40 minutes, the data for Hrq1-9-Myc suggest that Hrq1 is upregulated and then plateaus. For Hrq1 levels to drop again during the next cell cycle, one would expect degradation. It would be helpful to see this Western blot data extended to additional time points into the second cell cycle.

We agree with the reviewer that it would be ideal to analyze a second cell cycle. However, yeast cells lose their synchronization over time and therefore, we can only reliably analyze cells for one cell cycle.

Furthermore, given that the figure legends for Figures 1C and 2A clearly state that the experiment has been replicated, the fact that this information is missing from the legend in Figure 3A leads one to believe that these experiments have not been repeated. Thus, to make this claim that Hrq1 levels peak during S/G2, I believe that the data needs to

quantified, repeated, and extended to later time points, such that the return to G1 levels is clear.

All experiments, including **Figure 3a**, were repeated a minimum of three times (exception is the DRR assay where we had a technical issue with one of the replicates). We have now made that clear in the **Figure 3a** legend and quantified the data in the western blots from multiple experiments.

2) Figure 4D: This figure uses a recombination reporter assay previously published in Godin et al. 2013 NAR. Godin et al. contains a good schematic of this assay. A similar schematic should be provided in this figure.

We agree with the reviewer and have included a schematic of the recombination reporter assay used in **Figure 4d**.

Based on the diagram in Godin et al., it is clear that Leu+ colonies in this system can result from repair either through homologous recombination or single-strand annealing. These two possibilities can be distinguished by determining whether the resulting colonies are Ura+ or Ura-. The implications of these two outcomes for this study should be presented in the text. Moreover, the results of this analysis showing whether damage is repaired primarily through recombination or single-strand annealing should be included in the figure.

We have now included the rates of gene conversion and single-strand annealing observed upon over-expression of HRQ1 or HRQ1-7KR. We find that both gene conversion and single-strand annealing are elevated upon cisplatin exposure (**New Figure 4e**).

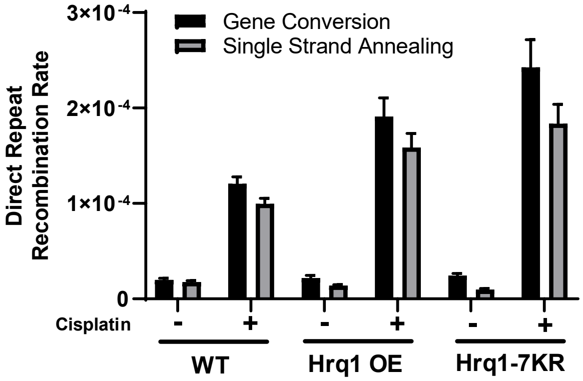

New Figure 4e Legend (4d is included for clarity)

d) Overexpression or stabilization of Hrq1 leads to increased recombination following cisplatin treatment (35 µg/ml, 16 hrs). Schematic of direct repeat recombination (DRR) assay, where recombinants are measured by formation of *LEU+* recombinants (graphed). In this assay, two *leu2* heteroalleles are disrupted by insertion of an EcoR1 or BsteII restriction sites, respectively, with an intervening *URA3* gene. Restoration of *LEU+* can occur by Rad51-dependent gene conversion (GC) measured by *URA3+ LEU2+* whereas Rad51-independent single-strand annealing (SSA) is measured by *URA3- LEU2+*. Nine independent colonies were measured for each experiment and the median value from two experiments (horizontal bar) were plotted. **

represents p<0.01, *** represents p<0.001 **e)** The average rates of GC and SSA for each condition are shown, average rates with SEM are graphed.

3) Figures 5C and D: Figures 5C and D: The manuscript does not show that the siRNA is specific to RECQL4 or REV1 by back complementation with siRNA resistant plasmids encoding RECQL4 or REV1. This is a relatively standard control for these types of experiments, and would create more confidence in the results, as it would show that the results are specific to RECQL4 and/or REV1.

We agree with the reviewer that using siRNA resistant plasmids to complement our assays are an important control. We tried to create a siRNA resistant plasmid for REV1, but we had difficulty cloning it. We also tried to buy an existing plasmid (https://www.addgene.org/153001/) to subclone into our vector but when we checked the gene sequence, the REV1 sequence was significantly truncated in the original plasmid. Unfortunately, due to the timing, we were not able to perform the REV1 complementation experiments. As for RECQL4, the siRNA sequence we used was from the Vilhelm Bohr's laboratory and has been used in multiple publications: Singh et al 2012, Ghosh et al 2012, Lu et al 2017.

4) Figure 6: A reasonable rationale for the use of U2OS cells to evaluate the effects of RECQL4 overexpression on DNA repair following cisplatin exposure is given. However, recently it has been shown by Löbrich and colleagues (Elbarky et al. 2021 PNAS) that U2OS specifically lack one of two recombination resolution sub-pathways, the ATRX-dependent double Holliday junction pathway, and predominantly use the RECQ5-dependent synthesis-dependent strand annealing (SDSA) pathway. This reliance on SDSA is not observed in HeLa and other cell lines. Thus, findings relating to homologous recombination in U2OS may not extend to other human cell lines/types, and this should be noted in the text as a potential shortcoming.

We thank the reviewer for this suggestion as we were unaware of the study by Löbrich and colleagues. We have now included discussion that use of SDSA as a predominant repair mechanism is not universal and cell line dependent in the Discussion.

5) Figure 6A: Quantifying only the total number of RAD51 foci present at a single time point is not an appropriate method to determine whether there is hyper-recombination in a given mutant. There are many alternative explanations that could explain the results observed, such as increased DNA damage, or RAD51 persistence due to unresolved recombination intermediates or lack of repair. The conclusions in this section should be revised to reflect this.

We agree with the reviewer and have revised the conclusions of this section to include these other possibilities as to why more RAD51 foci may be observed.

The authors should also indicate in their quantitation whether the difference in RAD51 foci between the empty vector and the RECQL4-KA mutant is statistically significant.

We have now included statistical analysis RAD51 foci compared with the empty vector and the RECQL4-KA mutant, and it is indeed statistically significant by t-test ($p < 0.01$).

6) Figure 7E: It has been reported that the majority of tumors overexpress RAD51 (e.g. Klein 2008 DNA Repair). Therefore, the implications of this finding are somewhat overstated, and should be revised.

We agree with the review and have now softened this statement.

7) Discussion: Please discuss how the findings of this study fit with other studies in the field, especially Lu et al. 2017 Nat Commun., since the conclusions differ from those in this study.

We have now included discussion of the Lu et al 2017 Nature Comms paper (p. 21). The primary difference between the Lu paper and our study is that they looked at RECQL4 roles during DSB repair, while our studies examined RECQL4 role during bypass of fork stalling lesions.

Additional Comments:

1) Methods: Under the Bioinformatic Analysis sub-section, Silver et al. 2010 is cited, which I believe refers to Silver et al. 2010 J Clin Oncol., as "(Silver et al. 2010)". This citation style is inconsistent with the rest of the manuscript. This error is made again in the Figure 7 legend. It should be corrected in both places. In addition, this reference should be added to the Bibliography at the end of the manuscript.

Corrected.

2) Figures 7F and G: The Methods section indicates that the analyses in these figures are based on results from a previously published study, Silver et al. 2010 (J of Clin Oncol.?). The study should be cited when referring to the findings in Figures 7F and G in the text to properly acknowledge it. It should also be noted that this study has a relatively small sample size (n = 24).

Corrected and sample size is now acknowledged.

3) Discussion: Throughout the Discussion, the manuscript refers to Figure 7 when I believe it intends to reference Figure 8.

Corrected.

4) Discussion: The manuscript states, "Furthermore, we find that Hrq1 is regulated by the UPS during instrastrand crosslink repair and that misregulation of Hrq1 protein levels results in increased recombination and mutagenesis. We extend this analysis to mammalian cells and show that like yeast, human RECQL4 protein levels are depleted in response to DNA crosslinks… Together our studies demonstrate a conserved role for the RECQL4 protein family in crosslink repair whose levels must be fine-tuned to prevent excess recombination and mutations." See comments on Figures 4 and 6 above. I believe the data

supporting these claims is relatively weak, and that this section should be revised to include more caveats and alternative explanations.

We agree with the reviewer and have modified this section accordingly.

5) Discussion: "Our studies here demonstrate that like RECQL4, Hrq1 also plays a key role during replication and is cell cycle regulated, peaking in the S/G2 cell cycle phase." It would be helpful if the authors could cite the original publications corresponding to the RECQL4 data and their own figures for the Hrq1 data.

Corrected.

Reviewer 3:

Major points
1. In Figure 1e, the authors report that a pulldown of His-tagged ubiquitin results in recovery of Hrq1 and conclude that Hrq1 is ubiquitylated. However, this experiment is missing key controls to demonstrate that the pulldown is specific for ubiquitylated species. This is especially important since the authors seem to recover large amounts on non-ubiquitylated Hrq1 in the pulldown. The authors should include a negative control such as a pulldown in the absence of of Cu2+ (when His-Ub is not expressed) or pulldown with agarose beads (no Ni) to confirm a specific pulldown. Additionally, the His-Ub pulldown protocol described in the methods section is incomplete (missing wash conditions).

We thank the reviewer for this helpful comment and have now repeated the pulldowns with the suggested controls. Unfortunately, the CUP1 promoter is leaky since there is a low level of copper in the medium. Therefore, even in the absence of cooper there is expression of the His-Ub. However, we are confident that we are pulling down 6x-His-Ub since we observe an enrichment of ubiquitin in the pulldown lanes (**Reviewer Figure 2**). In addition, we have included the washing conditions in the Methods section.

Reviewer Figure 2

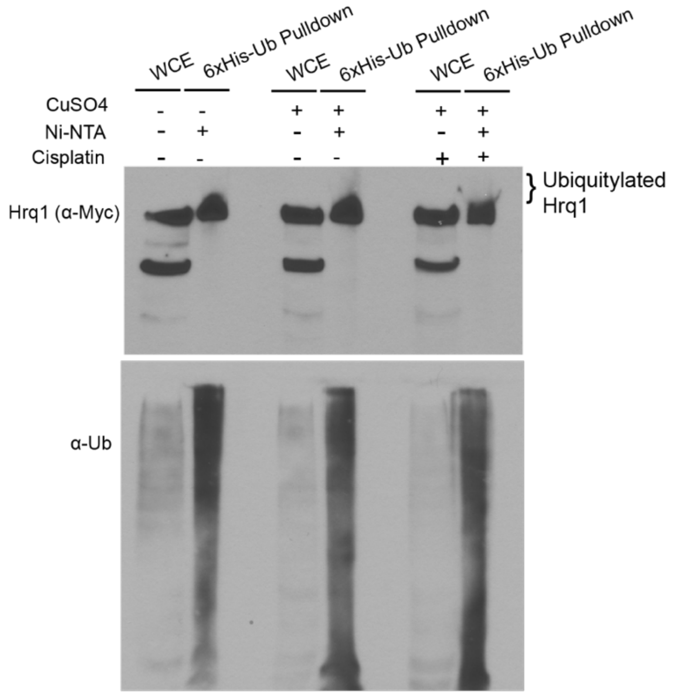

Reviewer Figure 2 Legend:
Ni-NTA pulldowns ubiquitylated proteins. Whole cell extract (WCE) or 6xHis-Ub pulldown was run on an 8% SDS-PAGE gel and blotted for Hrq1 (anti-Myc) or ubiquitin (anti-Ub) from uninduced (no copper sulfate) or copper sulfate induced cultures either untreated or treated with 100 µg/ml cisplatin. Two mM copper sulfate was added for 3 hrs, subsequently MG132 was added for an additional 45 mins followed by cisplatin for 45 min. In each condition, Ub was expressed, likely due to copper sulfate in the medium and/or a leaky CUP1 promoter.

2. Also concerning Figure 1e: the authors could strengthen their claim that the slowly migrating smear detected with the Hrq1 correspond to ubiquitylated Hrq1 by treating the recovered proteins with a general DUB such as USP21, which would be expected to collapse the putative ubiquitylated species into an unmodified form. As it stands, this reviewer does not find the authors' evidence that ubiquitylated Hrq1 accumulates upon cisplatin treatment very compelling.

We thank the reviewer for the suggestion, but this experiment is technically challenging since the His-Ub Hrq1 samples are denatured and we were unable to obtain a purified DUB. Therefore, we primarily focused on other reviewer concerns instead.

3. In Figure 1b, how do the authors confirm that treatment with MMC, HU, IR, and MMS were sufficient to elicit a robust DNA damage response? A trivial explanation is that the doses used for the experiment presented in 1b were too low to generate levels of DNA damage sufficient to induce Hrq1 degradation. Also, the doses used in 1b seem to be missing from the figure legend and/or methods.

We agree with the reviewer, and we inadvertently left off the doses analyzed, which are now included in **Figure 1b**. In addition, we have now performed additional experiments analyzing multiple doses (New Supplemental Figure 1b**, see Reviewer 1, Point 2**).

4. The authors propose a model in which Rad16-mediated degradation of Hrq1 constrains Hrq1 activity to allow for efficient resolution of post-replicative repair intermediates. Implicit in this model is an assumption that Hrq1 is ubiquitylated when associated with chromatin (Figure 8). Does deletion of Rad5 or Ubc13 prevent cisplatin-induced degradation of Hrq1? One might expect that blocking PCNA polyubiquitylation would prevent Hrq1 association with DNA and thereby block Hrq1 degradation.

We agree with the reviewer and find that *ubc13*Δ loss only mildly stabilizes Hrq1 protein levels upon cisplatin treatment by cycloheximide chase (**Supplemental Figure 2a**). We observed that blocking PCNA ubiquitylation did lead to a partial stabilization of Hrq1 following cisplatin exposure (**Supplemental Figure 2d**). This suggests that its degradation is in part in response to Hrq1 role during this process. However, Hrq1 is not fully stabilized suggesting that it may still be in at the chromatin. It is not known how poly-ubiquitination of PCNA recruit factors that mediate template switching, it is possible that Hrq1 is still at the chromatin in the absence of PCNA polyubiquitination.

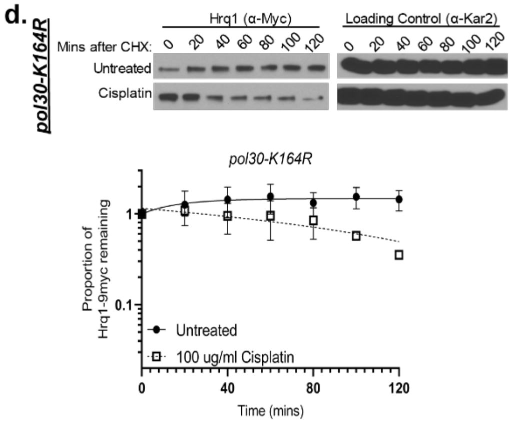

New Supplemental Figure 2d Legend
Hrq1-9xMYC expressing *pol30-K164R* cells were incubated with cycloheximide in the presence or absence of 100 μg/ml cisplatin and/or 0.1% DMSO. Protein extracts were analyzed by western blot for Hrq1 protein levels (α-MYC) or a loading control, Kar2 (α-Kar2), at the indicated time points.

5. Throughout the manuscript, the authors demonstrate that Hrq1 protein levels decline after treatment with cisplatin but not after treatment with MMC, MMS, IR, or HU. Cisplatin intrastrand cross-links are generally considered NER substrates while lesions formed by the other genotoxic agents are not. Is Hrq1 activity specifically directed toward NER substrates? This would be consistent with the authors' conclusion that Hrq1functions downstream of Ubc13 upon induction of DNA damage with UVC (another NER lesion). Are Hrq1 protein levels altered by UVC or BPDE? Are Δhrq1 cells hypersensitive to BPDE? Alternatively, cisplatin, aldehydes, and UV can all induce DNA-protein cross-links

(DPCs). Is there any evidence to suggest (or exclude) the possibility that Hrq1 participates in DPC-repair?

We thank the reviewer for this helpful suggestion and find that UVC exposure similarly results in Hrq1 degradation (**Supplemental Figure 3e**). To our knowledge, there is no evidence to suggest (or exclude) Hrq1 function in DPC-repair and this would be an interesting future investigation.

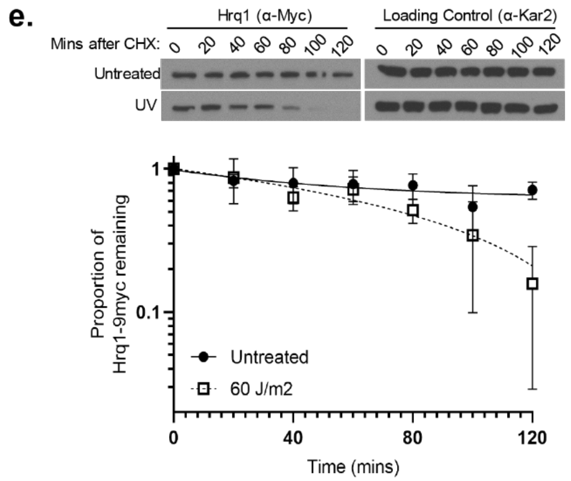

Supplemental Figure 3e Legend
Hrq1 protein levels decreased following UV-C treatment. Exponentially growing Hrq1-9xMYC cells were incubated with cycloheximide then treated with the indicated dosage of UV-C. Protein was extracted as described in **b**). Quantification from three separate experiments with mean and SEM is graphed.

6. The authors report that increased RECQL4 levels correlate with better tumor response to cisplatin treatment. Do cultured cells overexpressing RECQL4 (such as the U2OS cells described in Figure 6) exhibit hypersensitivity to cisplatin?

This is an important point and papers from Arora et al 2016 and Mo et al 2016demonstrated that RECQL4 over-expression does not result in cisplatin hypersensitivity in cultured TNBC and gastric cancer cell lines. These findings are in contrast to the clinical response to cisplatin measured from Silver et al 2010 where cisplatin was given as a neoadjuvant therapy in 24 TNBC tumors. It is possible that the increased mutations observed with RECQL4 over-expression elicits a tumor immune response that is not observed in culture or perhaps the small sample size or cohort analyzed may be distinct. We have now discussed these possibilities.

Minor points
1. The authors indicate that acetaldehyde “can produce similar lesions to cisplatin.” The cited literature (74) demonstrated that acetaldehyde can generate dG-dG intrastrand cross-links in a short single stranded oligonucleotide by cross-linking the N2 positions of dG. This lesion is distinct from the N7 cross-links formed by cisplatin. Is there any information as to how this lesion is repaired and whether this lesion forms in chromatin under the conditions

used in the experiments reported here? Also, the authors may consider parsing the chemistry of the various cross-links studied in order to orient a general reader.

The reviewer brings up important points regarding the different types of intrastrand crosslinks that can form with acetaldehyde and cisplatin. Unfortunately, we are not able to parse out the role of Hrq1 or RECQL4 in these specific lesions in the scope of this manuscript, but this would be interesting future direction using recombinant proteins.

2. On page 24, the authors report that loss of either UBC13 or RAD6 leads to only mild stabilization of Hrq1 following cisplatin treatment and suggest that additional E2 participate in Hrq1 ubiquitylation. Could UBC13 and RAD6 function redundantly to promote Hrq1 ubiquitylation? Have the authors attempted to measure Hrq1 stability following disruption of both UBC13 and RAD6?

We found deletion of both *UBC13* and *RAD6* genes did not lead to increased stability of Hrq1, suggesting that they cannot compensate for each other (**New Supplemental Figure 2c**).

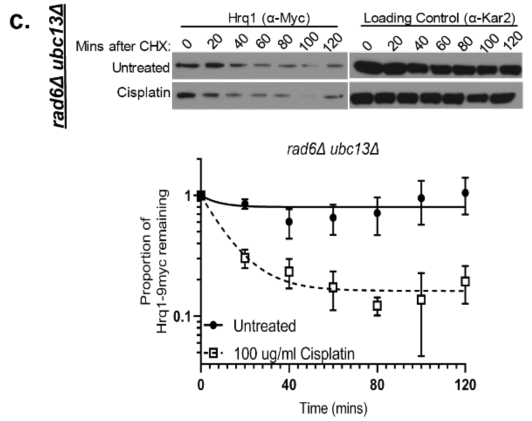

New Supplemental Figure 2c Legend

c) Hrq1-9xMYC expressing *rad6*Δ *ubc13*Δ cells were incubated with cycloheximide in the presence or absence of 100 µg/ml cisplatin and/or 0.1% DMSO. Protein extracts were analyzed by western blot for Hrq1 protein levels (α-MYC) or a loading control, Kar2 (α-Kar2), at the indicated time points.

3. The authors state: "We find that Hrq1/RECQL4 over-expression results in genomic instability." What is the basis for this statement, given that the authors show that RECQL4 overexpression fails to cause an increase in DSBs or ssDNA accumulation in Figure 6b.

Corrected to Hrq1.
